# Molecular Characterization of Soft Tissue Sarcomas Using RNA-Based Next-Generation Sequencing

**DOI:** 10.3390/ijms27062699

**Published:** 2026-03-16

**Authors:** Bogdan Serban, Adrian Cursaru, Sergiu Iordache, Bogdan Cretu, Mihai Nica, Georgian Iacobescu, Mihnea Popa, Eugen Radu, Madalina Cirnu, Catalin Cirstoiu

**Affiliations:** 1 Department of Orthopedics and Traumatology, University Emergency Hospital, 050098 Bucharest, Romania; bogdan.serban@umfcd.ro (B.S.); sergiu.iordache92@yahoo.com (S.I.); jfrbogdan@yahoo.com (B.C.); mikx99n@gmail.com (M.N.); georgian.iacobescu@umfcd.ro (G.I.); mihnea.popa@umfcd.ro (M.P.); eugen.radu@umfcd.ro (E.R.); madalina.cirnu@drd.umfcd.ro (M.C.); cirstoiu_catalin@yahoo.com (C.C.); 2 Orthopedics and Traumatology Department, Carol Davila University of Medicine and Pharmacy, 050474 Bucharest, Romania; 3 Molecular Biology and Pathology Research Laboratory, University Emergency Hospital, 050098 Bucharest, Romania

**Keywords:** sarcomas, RNA-NGS, gene expression

## Abstract

Soft tissue sarcomas are rare malignant mesenchymal tumors for which accurate diagnosis, prognostic stratification, and therapeutic decision-making remain challenging. Although histopathology and immunohistochemistry are essential diagnostic tools, they frequently fail to capture the molecular complexity underlying tumor aggressiveness and treatment resistance. In this study, we evaluated the utility of RNA-based next-generation sequencing for the molecular characterization of STS and for elucidating transcriptomic mechanisms associated with aggressive tumor behavior. An observational cohort of 24 patients with histologically confirmed soft tissue sarcomas was analyzed, using adipose and skeletal muscle tissue as controls. RNA was extracted from tumor samples, libraries were prepared with a targeted pan-cancer panel, and sequencing was performed on the Illumina platform, followed by bioinformatic analysis using DRAGEN pipelines and DESeq2. RNA-NGS identified a predominance of single-nucleotide polymorphisms and significant differential gene expression, with overexpression of proliferation-related genes (*TOP2A*, *MKI67*, *BUB1B*), extracellular matrix and microenvironment-associated genes (*COL11A1*, *SPP1*), and developmental regulators (*HOXD13*, *MELK*). Subgroup analysis revealed a distinct transcriptomic profile in leiomyosarcoma, while gene fusion analysis detected clinically relevant alterations. These findings demonstrate that RNA-NGS provides biologically and clinically meaningful insights into the molecular landscape of soft tissue sarcomas and supports its integration into precision medicine-oriented diagnostic workflows.

## 1. Introduction

Soft tissue sarcomas (STS) constitute a rare and biologically diverse group of malignant mesenchymal neoplasms, accounting for approximately 1% of all adult malignancies [1]. Their marked heterogeneity extends beyond histological variability to encompass complex genetic and phenotypic alterations, posing substantial challenges to accurate diagnosis, prognostic stratification, and therapeutic decision-making. While histopathological examination supplemented by immunohistochemistry (HP/IHC) remains the diagnostic cornerstone, these conventional approaches often fail to adequately reflect the underlying molecular complexity of STS, particularly in tumors with overlapping morphological characteristics or inconclusive immunophenotypic profiles.

Building on our previous work demonstrating the prognostic relevance of tumor margin infiltration and immune markers in soft tissue sarcomas, the present study explores the underlying molecular and transcriptomic alterations using RNA-based next-generation sequencing [2].

Over the past two decades, advances in molecular biology have significantly expanded the understanding of sarcoma pathogenesis, prompting the integration of molecular alterations into the current World Health Organization (WHO) classification [3]. Nevertheless, the routine implementation of molecular diagnostics in clinical practice remains limited and standardized molecular algorithms for diagnostic and prognostic evaluation of STS have yet to be established. As a result, therapeutic strategies frequently rely predominantly on clinicopathological criteria, despite the modest efficacy of conventional systemic treatments in patients with locally advanced or metastatic disease.

Within this framework, next-generation sequencing (NGS) technologies have emerged as powerful platforms for comprehensive molecular profiling, allowing simultaneous assessment of gene expression patterns, single-nucleotide variants, insertions and deletions, and oncogenic gene fusions. RNA-based NGS (RNA-NGS), in particular, offers a distinct advantage by capturing transcriptionally active alterations, including fusion events and deregulated signaling pathways with potential diagnostic, prognostic, or therapeutic relevance [4].

Although RNA-based NGS is increasingly adopted in specialized centers—especially for fusion detection and diagnostic refinement—evidence supporting its routine, nonselective use across all STS entities and its standardized prognostic implementation is still evolving, and results should be interpreted within multidisciplinary sarcoma-expert settings. Accordingly, the present study aimed to explore the feasibility and transcriptomic landscape of STS using RNA-based NGS, with a focus on identifying biologically relevant expression patterns and clinically meaningful fusion events within a real-world cohort.

## 2. Results

The sequencing data were manually analyzed using DESeq2, based on which three types of analyses were performed using volcano plots and heatmaps (with the exception of Analysis C, as the analyzed sample cohort was not sufficiently large to allow robust discrimination of differentially expressed genes between the compared variants).

### 2.1. Analysis A

**Figure 1:**
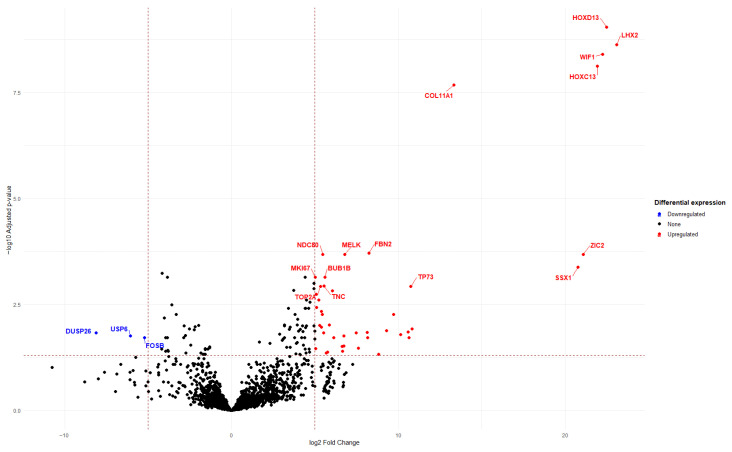
Molecular analysis of soft tissue sarcomas versus control samples. Volcano plot depicting differential gene expression between STS and control tissues (adipose and skeletal muscle). Differential expression was computed in DESeq2 using size-factor normalized counts (Wald test). *p*-values were adjusted for multiple testing using Benjamini–Hochberg FDR; the *y*-axis shows –log10(adjusted *p*-value), and the *x*-axis shows log2 fold change.

To evaluate whether global transcriptomic profiling distinguishes among sarcoma subtypes, we performed unsupervised principal component analysis (PCA) using variance-stabilized normalized expression values (Figure 1).

The first two principal components accounted for 41% of total variance. Leiomyosarcoma samples demonstrated moderate separation from other sarcoma subtypes along PC1/PC2 axes. However, overlapping clusters were observed among certain entities, reflecting shared proliferative and oncogenic signatures across high-grade sarcomas.

Hierarchical clustering analysis based on variance-stabilized expression data showed predominant grouping by histologic subtype, although partial intermixing was observed among high-grade tumors

The results of the volcano plot analysis illustrate gene expression changes as a function of the statistical significance of differential expression, as follows:

- *X*-axis (Log2 Fold Change) represents the magnitude of gene expression change between the two compared groups (tumor vs. control). Positive values indicate upregulated genes (higher expression in tumor samples), whereas negative values indicate downregulated genes (lower expression in tumor samples).
- *Y*-axis (Log10 adjusted *p*-value) represents the statistical significance of gene expression differences. Higher values indicate greater statistical significance.
- Dashed threshold lines define quadrants used to determine both statistical significance and expression level. Genes located above the horizontal dashed line are considered statistically significant (adjusted *p* < 0.05), while genes located beyond the vertical dashed lines are considered markedly differentially expressed between the two groups (Fold Change < −5 or >5).

Genes located in the upper left and upper right regions of the plot are the most relevant, as they combine high statistical significance with large expression changes.

**Figure 2:**
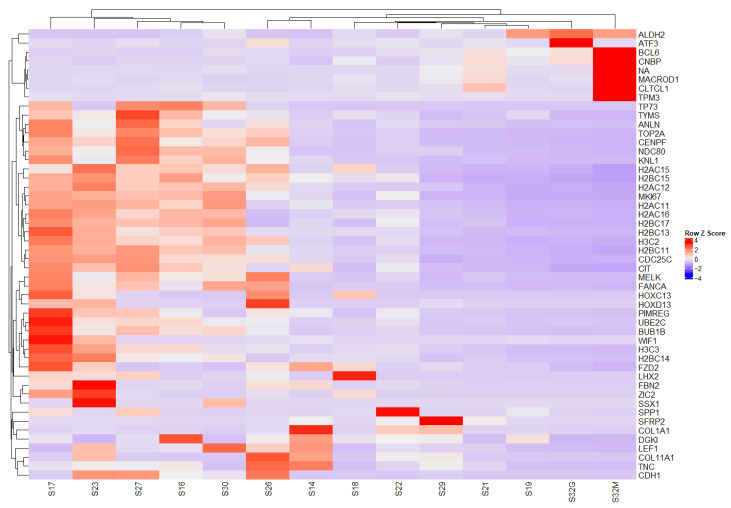
Expression and clustering of the top 50 differentially expressed genes. Soft tissue sarcomas versus control. Heatmap of the top 50 DEGs between STS and controls. Values represent variance-stabilized transformed (VST) counts displayed as row-wise Z-scores. Clustering was performed using Euclidean distance and complete linkage on variance-stabilized (VST) normalized expression values.

Accordingly, the most highly overexpressed genes identified in Analysis A were: *TOP2A*, *TNC*, *BUB1B*, *MKI67*, *NDC80*, *MELK*, *FBN2*, *TP73*, *SSX1*, *ZIC2*, *COL11A1*, *HOXC13*, *WIF1*, *LHX2*, and *HOXD13* [Figure 2].

Across the analyzed samples, the total number of detected variants ranged from 620 to 1377 per sample, with SNPs representing the predominant variant class.

The transition/transversion (Ti/Tv) ratios ranged between 3.13 and 3.69, consistent with expected transcriptomic variant distributions in high-quality RNA sequencing datasets.

Heterozygous to homozygous variant ratios ranged between 0.51 and 1.55, reflecting inter-sample genomic heterogeneity.

Heatmap analysis displays gene expression levels in a matrix format, where each cell represents the expression level of a specific gene in a given sample. Red indicates higher gene expression, whereas blue indicates lower expression levels, with color intensity reflecting relative expression magnitude.

- Notably, this heatmap depicts gene-by-gene expression across all samples only after differential expression analysis (tumor vs. control) had been performed.
- The dendrograms displayed above and to the left of the heatmap cluster samples and genes based on expression similarity.
- This visualization facilitates the simultaneous comparison of multiple samples, enabling the identification of gene clusters with similar expression profiles and the recognition of expression patterns characteristic of specific tumor conditions or subtypes.

The expression and clustering of the top 50 differentially expressed genes highlighted the presence of *BCL6*, *CNBP*, *NA*, *MACROD1*, *CLTCL1*, *TPM3*, *SSX1*, *SPP1*, *COL1A1*, *LHX2*, *PIMREG*, *FANCA*, *MELK*, *CDC25C*, *HOXD13*, and *MKI67*.

### 2.2. Analysis B

**Figure 3:**
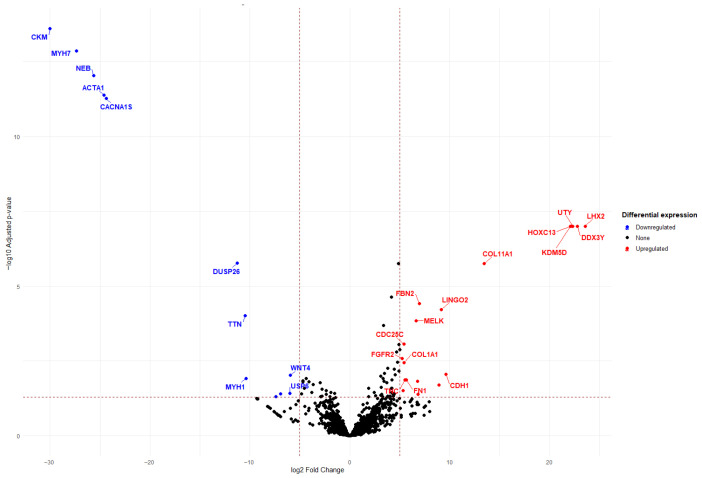
Molecular analysis of leiomyosarcoma versus control samples. Volcano plot depicting differential gene expression between leiomyosarcoma and control samples, performed using DESeq2, applying a negative binomial generalized linear model (Wald test). *p*-values were adjusted for multiple testing using the Benjamini–Hochberg false discovery rate (FDR) method.

Differential expression was computed using DESeq2 with a Wald test and Benjamini–Hochberg FDR correction (Figure 3).

**Figure 4:**
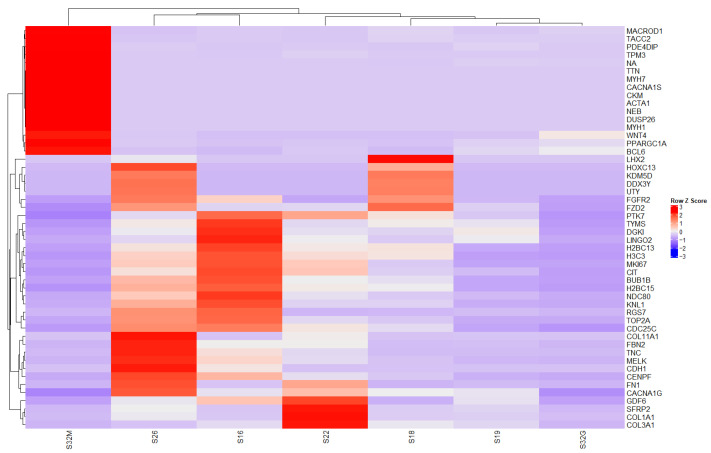
Expression and clustering of the top 50 differentially expressed genes. Leiomyosarcoma versus control. Heatmap of the top 50 DEGs for leiomyosarcoma versus controls using VST counts displayed as row-wise Z-scores with unsupervised hierarchical clustering.

The results of Analysis B (leiomyosarcoma vs. control) (Figure 4) demonstrated significant overexpression of *FN1*, *CDH1*, *FGFR2*, *CDC25C*, *MELK*, *LINGO2*, *FBN2*, *COL11A1*, *KDM50*, *HOXC13*, *UTY*, *LHX2*, *DDX3Y*, and *HOXC13*. Marked overexpression of the most differentially expressed genes was further evidenced by the presence of *MACROD1*, *TACC2*, *PDE4DIP*, *TPM3*, *NA*, *TTN*, *MYH7*, *CACNA1S*, *CKM*, *ACTA1*, *UTY*, *FGFR2*, *PTK7*, *TOP2A*, and *KNL1*.

### 2.3. Analysis C

**Figure 5:**
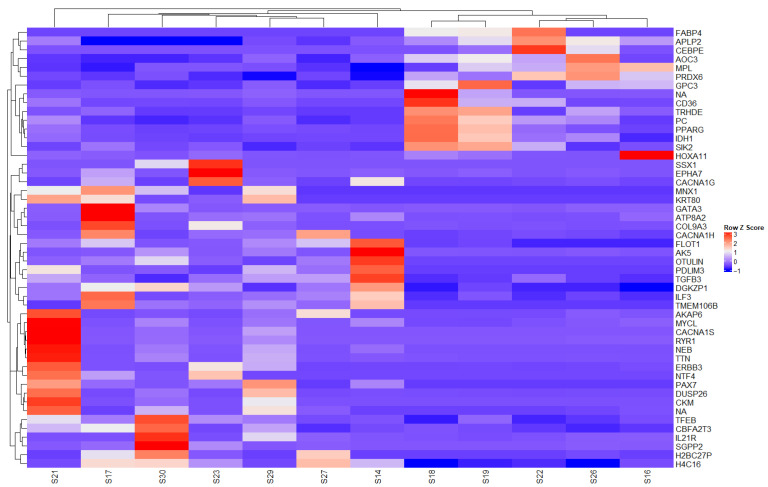
Exploratory heatmap comparing leiomyosarcoma to other STS subtypes. Values represent VST counts displayed as row-wise Z-scores. This analysis is descriptive given limited subgroup sample size.

In Analysis C, the most highly overexpressed genes identified were *AKAP6*, *MYCL*, *CACNA1S*, *RYR1*, *NEB*, *HOXA11*, *NA*, *CD36*, *MYNX1*, *KRT80*, and *IL21R* (Figure 5).

### 2.4. Integrated Overview of Molecular Findings

Collectively, RNA-based next-generation sequencing revealed a complex transcriptomic landscape characterized by deregulation of genes involved in proliferation, mitotic control, extracellular matrix remodeling, and developmental signaling. These molecular alterations were observed across the entire cohort and within specific sarcoma subtypes, highlighting both shared and subtype-specific transcriptional programs underlying soft tissue sarcoma biology.

In addition to overexpressed transcripts, we identified significantly downregulated genes (adjusted *p*-value < 0.05; log2 fold change < −5).

Among the most significantly underexpressed genes were *CKM*, *MYH7*, *NEB* and *ACTA1*, which are involved in structural maintenance and muscle differentiation.

These findings suggest that sarcoma progression may involve not only proliferative activation but also suppression of tissue differentiation programs and structural regulatory pathways.

RNA-based fusion detection identified multiple fusion events across the cohort. The canonical *SS18::SSX1* fusion was detected in synovial sarcoma samples (confidence score 1.000; 6 paired reads; 45 split reads), confirming the diagnostic robustness of the RNA-NGS approach.

Additional recurrent fusions included *HMGA2::C9orf16* (score 0.933–0.995; up to 88 paired reads and 62 split reads) and *FRS2::MDM2* (score 0.962; 24 paired reads; 15 split reads).

The detection of these alterations highlights the ability of targeted RNA sequencing to capture clinically relevant fusion events and amplification-associated rearrangements within soft tissue sarcomas.

## 3. Discussion

Soft tissue sarcomas (STS) are characterized by remarkable biological heterogeneity, reflected in diverse transcriptional programs that drive tumor initiation, progression, and therapeutic resistance. In the present study, RNA-based next-generation sequencing enabled comprehensive transcriptomic profiling and fusion detection, revealing consistent overexpression of genes involved in cell cycle regulation, proliferation, extracellular matrix remodeling, and developmental signaling pathways. These findings support the concept that STS progression is driven by convergent molecular mechanisms despite histological diversity.

A prominent finding of this analysis was the consistent overexpression of proliferation-associated genes, including *TOP2A*, *MKI67*, and *BUB1B*. Overexpression of *TOP2A* has been widely reported in high-grade soft tissue sarcomas, particularly leiomyosarcoma and undifferentiated pleomorphic sarcoma, and is associated with increased mitotic activity, genomic instability, and poor clinical outcomes [5]. Importantly, *TOP2A* expression has been correlated with response to anthracycline-based chemotherapy, suggesting potential predictive value in treatment selection [Table 1].

This exploratory study used real-world transcriptomic analysis in a clinically managed STS cohort. Due to the rarity and heterogeneity of soft tissue sarcomas, large homogeneous datasets are challenging to obtain at single centers. The main goal was to assess the feasibility and RNA-based NGS transcriptomic patterns, not to establish prognostic biomarkers. This approach reflects the current stage of molecular profiling in STS.

Similarly, *MKI67* overexpression reflects high proliferative activity and has been shown to correlate with FNCLCC grade, metastatic potential, and reduced overall survival across multiple sarcoma subtypes, including leiomyosarcoma and synovial sarcoma [6]. These data indicate that transcriptomic assessment of proliferation markers may provide a more objective and quantitative measure of tumor aggressiveness compared to conventional histopathological evaluation alone.

*BUB1B*, a key regulator of the mitotic spindle checkpoint, was also significantly overexpressed. Dysregulation of *BUB1B* has been linked to chromosomal instability and aggressive tumor behavior in sarcomas with complex karyotypes, particularly leiomyosarcoma and pleomorphic sarcoma [7]. Its expression underscores the role of mitotic checkpoint failure as a driver of sarcoma progression. In addition to proliferation-related genes, extracellular matrix (ECM)-associated genes were prominently deregulated. *COL11A1* emerged as one of the most consistently overexpressed genes, in line with previous reports identifying it as a marker of activated tumor stroma in leiomyosarcoma, fibrosarcoma, and desmoplastic tumors [8]. *COL11A1* overexpression has been associated with increased invasiveness, resistance to therapy, and unfavorable prognosis, highlighting the importance of tumor–stroma interactions in STS biology.

The overexpression of *SPP1* (osteopontin) further supports the role of the tumor microenvironment in sarcoma aggressiveness. *SPP1* has been linked to enhanced cell migration, angiogenesis, and metastatic potential in multiple sarcoma subtypes, and its expression correlates with poor survival outcomes [9]. Together, these findings suggest that ECM remodeling and microenvironmental signaling are critical contributors to sarcoma progression.

Developmental and transcriptional regulators were also deregulated. *HOXD13*, a member of the HOX gene family, has been implicated in aberrant mesenchymal differentiation and developmental pathway reactivation in sarcomas. Overexpression of *HOXD13* has been described in poorly differentiated sarcomas and is associated with loss of normal tissue identity and increased tumor aggressiveness [10]. These findings reinforce the concept that sarcomas exploit embryonic transcriptional programs to sustain malignant behavior.

The identification of *SSX1* overexpression and the detection of the canonical *SS18::SSX1* fusion in synovial sarcoma samples validate the robustness of the RNA-NGS approach. This fusion is pathognomonic for synovial sarcoma and plays a central role in epigenetic dysregulation and transcriptional reprogramming [11]. Beyond its diagnostic value, emerging evidence suggests that the *SS18–SSX* fusion complex represents a potential therapeutic vulnerability, further emphasizing the clinical relevance of molecular profiling.

Additional deregulated genes, including *MELK*, *TPM3*, and *BCL6*, further illustrate the molecular complexity of STS. *MELK* overexpression has been associated with stemness, proliferation, and therapeutic resistance in high-grade sarcomas, positioning it as a potential target for kinase inhibition (IJMS). *TPM3* has been implicated in cytoskeletal organization and cell motility and is frequently involved in fusion-driven mesenchymal tumors. Meanwhile, aberrant *BCL6* expression may contribute to transcriptional repression and apoptosis evasion, supporting tumor survival in aggressive sarcoma subtypes.

Collectively, these results demonstrate that RNA-based NGS provides clinically meaningful insights into the molecular landscape of soft tissue sarcomas, enabling simultaneous evaluation of gene expression patterns and oncogenic fusions. The integration of transcriptomic profiling into routine diagnostic workflows may refine sarcoma classification, improve prognostic stratification, and identify novel therapeutic targets, supporting the implementation of precision oncology approaches in this challenging group of malignancies.

Importantly, transcriptomic alterations were not limited to proliferative gene activation. Several genes exhibited marked downregulation across tumour samples. Although RNA sequencing cannot directly detect structural gene inactivation at the DNA level, consistent transcriptional suppression may reflect gene silencing, epigenetic alterations, or tumour dedifferentiation processes.

Future integrative analyses combining RNA-based profiling with DNA-level genomic assessment would help determine whether these downregulated genes correspond to structural alterations, copy number loss, or regulatory suppression mechanisms.

## 4. Materials and Methods

### 4.1. Study Cohort

This observational cohort study included 24 patients diagnosed with soft tissue sarcomas based on histopathological and immunohistochemical evaluation. Tumor samples were obtained from patients undergoing either incisional biopsy or definitive surgical excision. A control group consisting of adipose and skeletal muscle tissue samples was included for comparative molecular analysis. All patients were managed according to institutional diagnostic and therapeutic protocols, and clinical data were collected prospectively.

Total RNA input per sample ranged between 11.2–12.2 ng, with RNA integrity numbers (RIN) ≥ 5 and a DV200 value of ≥50% in all included specimens. Libraries were prepared using the TruSight RNA Pan-Cancer Panel (Illumina) according to the manufacturer’s protocol.

Sequencing was performed on an Illumina MiniSeq platform using paired-end chemistry (2 × 75 bp).

The mean sequencing depth was 2.8 million reads per sample (range: 1.4–3.25 million reads). The mean target coverage achieved across samples was 403× (range: 226×–471×).

Quality metrics demonstrated high sequencing performance, with a mean Q30 percentage of 90.01% (range: 87.87–90.77%).

Variant-level quality metrics showed transition/transversion (Ti/Tv) ratios ranging between 3.1–3.7, consistent with high-quality RNA sequencing data.

No samples were excluded following quality control filtering.

The cohort comprised 24 STS cases, including leiomyosarcoma (*n* = 4), synovial sarcoma (*n* = 4), liposarcoma (*n* = 4), Undifferentiated pleomorphic sarcoma (*n* = 4), angiosarcoma (*n* = 2), rhabdomyosarcoma (*n* = 2), fibrosarcoma (*n* = 2), and epithelioid sarcoma (*n* = 2). The control group included adipose tissue (*n* = 2) and skeletal muscle tissue (*n* = 2), processed using the same protocol.

#### 4.1.1. Inclusion Criteria

(i) Histopathologically and immunohistochemically confirmed STS;
(ii) Availability of sufficient tissue for RNA extraction;
(iii) RNA passing minimal quality/quantity thresholds for library preparation;
(iv) Availability of clinicopathologic classification (histologic subtype).

#### 4.1.2. Exclusion Criteria

(i) Insufficient material;
(ii) Failure of RNA extraction/QC;
(iii) Extensively necrotic/hemorrhagic tissue precluding representative sampling;
(iv) Incomplete clinicopathologic information preventing subtype assignment.

Controls: adipose and skeletal muscle tissues processed using the same pre-analytical workflow. Control samples with inadequate RNA QC or marked tissue degradation were excluded.

### 4.2. RNA Extraction and Quantification

Tumor and control tissue samples were preserved in RNAlater solution immediately after collection to ensure RNA integrity. Total RNA was extracted using the RNeasy Fibrous Tissue Mini Kit (QIAGEN—Hilden, Germany), following the manufacturer’s instructions. RNA concentration and quality were assessed using the Qubit RNA High Sensitivity Assay (Thermo Fisher Scientific), ensuring suitability for downstream library preparation and sequencing.

### 4.3. Library Preparation and Sequencing

RNA sequencing libraries were prepared using the TruSight RNA Pan-Cancer Panel (Illumina) according to the manufacturer’s protocol and sequenced on an Illumina MiniSeq using paired-end chemistry (2 × 75 bp). For library preparation, 11.2–12.2 ng of total RNA was used per sample. Sequencing achieved a mean depth of 2.8 million reads per sample (range: 1.4–3.25 million reads).

### 4.4. Bioinformatic Analysis

Sequencing data were processed using Illumina’s cloud-based BaseSpace environment. Primary analysis was conducted using the DRAGEN RNA Pipeline version 4.2.7 for variant calling, including single-nucleotide polymorphisms (SNPs), insertions/deletions (InDels), and gene fusion detection. Additional alignment and expression analyses were performed using the RNA-Seq Alignment workflow version 2.0.2. Differential gene expression analysis was carried out using DESeq2 v1.43.3, enabling the identification of significantly deregulated genes between tumor and control samples, as well as within specific sarcoma subgroups.

## 5. Conclusions and Limitations

### 5.1. Conclusions

This study demonstrates that RNA-based next-generation sequencing represents a robust and clinically relevant approach for the molecular characterization of soft tissue sarcomas. By integrating transcriptomic profiling with fusion gene detection, RNA-NGS enabled the identification of key molecular alterations associated with tumor proliferation, extracellular matrix remodeling, and aberrant developmental signaling pathways.

The consistent overexpression of proliferation-related genes (*TOP2A*, *MKI67*, *BUB1B*), stromal and microenvironment-associated genes (*COL11A1*, *SPP1*), and transcriptional regulators (*HOXD13*, *MELK*, *BCL6*) highlights convergent biological mechanisms underlying sarcoma aggressiveness despite marked histological heterogeneity. Furthermore, the detection of clinically relevant gene fusions, including the canonical *SS18::SSX1* fusion in synovial sarcoma and collagen-related fusions in fibrous tumors, underscores the diagnostic accuracy and added value of RNA-NGS beyond conventional histopathology and immunohistochemistry.

Overall, these findings provide a transcriptomic framework that may contribute to improved molecular understanding of STS biology and support future validation studies. While RNA-based NGS demonstrates clear diagnostic and biological value, further prospective validation is required before broad implementation in routine prognostic or therapeutic decision-making can be recommended.

### 5.2. Limitations

First, orthogonal validation of selected DEGs (e.g., RT-qPCR and/or immunohistochemistry) was not performed in the current study, and therefore, our transcriptomic findings should be interpreted as hypothesis-generating and require independent validation.

Consequently, the differentially expressed genes identified in this study should be regarded as candidate transcriptomic markers rather than validated molecular targets. Independent validation using orthogonal techniques such as RT-qPCR, immunohistochemistry, and functional assays in larger prospective cohorts will be necessary before clinical implementation can be considered.

Several limitations of the present study should be acknowledged.

First, the relatively small sample size and the inherent heterogeneity of soft tissue sarcoma subtypes may limit the generalizability of the findings.

Second, the retrospective nature of the analysis precluded a direct assessment of the predictive value of the identified molecular alterations with respect to treatment response and long-term survival outcomes. Additionally, functional validation of the deregulated genes and fusion events was not performed, and further experimental studies are required to elucidate their precise biological roles.

Despite these limitations, the study provides valuable real-world molecular data and supports the feasibility and clinical relevance of RNA-based next-generation sequencing in the diagnostic and translational research setting of soft tissue sarcomas.

Although RNA sequencing provides valuable insights into transcriptional alterations and fusion detection, it does not capture structural genomic rearrangements, copy number variations, or epigenetic modifications. Therefore, certain mechanisms of gene inactivation may remain undetected using this approach alone.

## Figures and Tables

**Table 1:** Key differentially expressed genes in soft tissue sarcomas and their associated biological pathways and clinical relevance.

Gene	Associated Sarcoma Subtypes	Biological Role	Associated Signaling Pathway	Clinical Relevance
*TOP2A*	Leiomyosarcoma, UPS, high-grade STS	Cell cycle progression, DNA replication	Cell cycle regulation/DNA replication machinery	Marker of proliferation, poor prognosis, potential predictor of anthracycline response
*MKI67*	Leiomyosarcoma, synovial sarcoma, UPS	Cellular proliferation	Proliferation signaling/cell cycle control	Correlates with tumor grade, metastatic risk, reduced OS
*BUB1B*	Leiomyosarcoma, pleomorphic sarcoma	Mitotic checkpoint control	Mitotic checkpoint signaling	Genomic instability, aggressive behavior
*COL11A1*	Leiomyosarcoma, fibrosarcoma, desmoplastic STS	Extracellular matrix remodeling	ECM organization/TGF-β-related stromal signaling	Invasiveness, stromal activation, poor prognosis
*SPP1*	High-grade STS, metastatic sarcomas	Cell migration, angiogenesis	Integrin-mediated signaling/PI3K–AKT pathway	Metastatic potential, unfavorable outcome
*SSX1*	Synovial sarcoma	Transcriptional dysregulation (via fusion)	Epigenetic remodeling/chromatin regulation	Diagnostic marker, therapeutic target potential
*HOXD13*	Poorly differentiated STS, LMS, synovial sarcoma	Developmental signaling	HOX developmental signaling pathway	Loss of differentiation, aggressive phenotype
*MELK*	Leiomyosarcoma, high-grade STS	Cell cycle regulation, stemness	MAPK-related signaling/proliferation control	Therapy resistance, emerging drug target
*TPM3*	Fibroblastic STS, fusion-driven tumors	Cytoskeletal organization	Actin cytoskeleton dynamics	Cell motility, invasion
*BCL6*	Aggressive STS subtypes	Transcriptional repression	Transcriptional regulatory networks	Apoptosis evasion, poor survival

## Data Availability

The data presented in this study are only available on request from the corresponding author due to ethical reasons. The data provided were part of the phd thesis. https://umfcd.ro/teze-de-doctorat-sustinute/sustinere-teza-drd-serban-g-bogdan/, accessed date: 5 March 2026.

## Abbreviations

STS	soft tissue sarcoma
RNA-NGS	RNA-based next-generation sequencing
DEG	differentially expressed gene
log2FC	log2 fold change
FDR	false discovery rate
PCA	principal component analysis
VST	variance-stabilizing transformation
IHC	immunohistochemistry
RT-qPCR	reverse transcription quantitative PCR
SNV	single-nucleotide variant
Indel	insertion/deletion
